# Understanding how primary care practitioners perceive an online intervention for the management of hypertension

**DOI:** 10.1186/s12911-016-0397-x

**Published:** 2017-01-09

**Authors:** Katherine Bradbury, Katherine Morton, Rebecca Band, Carl May, Richard McManus, Paul Little, Lucy Yardley

**Affiliations:** 1 Academic unit of psychology, University of Southampton, Southampton, UK; 2 Faculty of Health Sciences, University of Southampton, Southampton, UK; 3 Nuffield Department of Primary Care Health Sciences, University of Oxford, Oxford, UK; 4 Primary Care and Population Sciences, Faculty of Medicine, University of Southampton, Southampton, UK

**Keywords:** Person-based approach, Qualitative research, Intervention development, Hypertension

## Abstract

**Background:**

In order to achieve successful implementation an intervention needs to be acceptable and feasible to its users and must overcome barriers to behaviour change. The Person-Based Approach can help intervention developers to improve their interventions to ensure more successful implementation. This study provides an example of using the Person-Based Approach to refine a digital intervention for hypertension (HOME BP).

**Methods:**

Our Person-Based Approach involved conducting qualitative focus groups with practice staff to explore their perceptions of HOME BP and to identify any potential barriers to implementation of the HOME BP procedures. We took an iterative approach moving between data collection, analysis and modifications to the HOME BP intervention, followed by further data collection. The data was analysed using thematic analysis.

**Results:**

Many aspects of HOME BP appeared to be acceptable, persuasive and feasible to implement. Practitioners perceived benefits in using HOME BP, including that it could empower patients to self-manage their health, potentially overcome clinical inertia around prescribing medication and save both the patient and practitioner time. However, practitioners also had some concerns. Some practitioners were concerned about the accuracy of patients’ home blood pressure readings, or the potential for home monitoring to cause patients anxiety and therefore increase consultations. Some GPs lacked confidence in choosing multiple medication changes, or had concerns about unanticipated drug interactions. A few nurses were concerned that the model of patient support they were asked to provide was not consistent with their perceived role. Modifications were made to the intervention based on this feedback, which appeared to help overcome practitioners’ concerns and improve the acceptability and feasibility of the intervention.

**Conclusions:**

This paper provides a detailed example of using the Person-Based Approach to refine HOME BP, demonstrating how we improved the acceptability and feasibility of HOME BP based on feedback from practice staff. This demonstration may be useful to others developing digital interventions.

## Background

High blood pressure increases the chances of cardiovascular disease and stroke [1] and is the leading risk factor for global disease burden [2]. Despite the availability of a range of effective medications for treating hypertension, control and treatment in the UK is currently suboptimal [3]. This is primarily because clinicians do not intensify treatment in response to raised blood pressure [4], and patients do not adhere to medication and behavioural self-management [5].

Patient self-monitoring of blood pressure at home and implementation of pre-agreed medication changes is a highly effective way of managing hypertension, as demonstrated by the TASMINH2 and TASMIN-SR studies which applied these procedures in a UK Primary Care context, employing face-to-face training for patients and practitioners [6, 7]. These two randomised controlled trials showed that compared to usual care, self-monitoring of blood pressure at home and implementation of pre-agreed medication changes led to significantly greater reductions in blood pressure in patients with hypertension [6], and cardiovascular disease, diabetes or chronic kidney disease [7]. Online delivery could provide a cost-effective way of automating the TASMIN procedures, so that this intervention could be rolled out more widely. We recently adapted the TASMIN methodology for online delivery through a digital intervention named HOME BP [8]. Additionally, support for making healthy behaviour changes (e.g. diet, physical activity) was also included in HOME BP, since such changes are also recommended to reduce blood pressure [9].

When carrying out face-to-face training, as used in the TASMIN studies, the trainer is made aware if patients or practitioners do not understand or have concerns about a procedure and can therefore immediately adapt the training on the spot to overcome these barriers to implementation. This is of course not possible in automated online interventions, so instead great care must be taken to ensure that the online training is acceptable and potential barriers to implementation are overcome in advance of training end users. The Person-Based Approach provides a method for ensuring that online interventions are as acceptable, engaging, persuasive and feasible to carry out as possible, to help ensure effective implementation [10]. Initially, during the intervention planning phase, the Person-Based Approach involves drawing on qualitative work exploring target users’ views and needs in order to formulate ‘guiding principles’ which are brief summaries of the distinctive ways in which the intervention will address key context-specific behavioural issues. The development of guiding principles for HOME BP is reported in detail elsewhere [Band, Bradbury, Morton, May, Michie, Mair, Murray, McManus, Little, Yardley. Intervention planning for a digital intervention for self-management of hypertension: a Theory-, Evidence-, and Person-Based Approach. Submitted]. The guiding principles identified in the planning stage are used to guide the development of a prototype intervention. Once a prototype is available the intervention development phase begins, which involves gaining detailed feedback on the prototype. This paper presents a detailed example of how we used the Person-Based Approach during a development phase, to refine our prototype version of HOME BP; this may serve as a useful demonstration of the approach to others interested in developing digital interventions.

Our Person-Based approach to intervention development involved carrying out iterative qualitative work with patients [Bradbury, Grist, Morton, Band, McManus, Little, Yardley. Patients’ perceptions of the HOME BP intervention. Unpublished] and GP practice staff who viewed our prototype version of HOME BP. This approach involved moving in cycles between data collection, analysis of users’ accounts to identify possible barriers to behaviour change and implementation of procedures, modifications to the website to help overcome identified barriers, then further data collection to assess the impact of our changes. This article reports the qualitative study with GP Practice staff who took part in focus groups to provide feedback on the HOME BP intervention. The aim of this study was to examine how practice staff perceived the HOME BP intervention, in particular we were interested in how acceptable and feasible the intervention might be to implement in practice and what further modifications might be needed to optimise the intervention for practice staff.

## Methods

### Design

We used focus groups to elicit qualitative data in order to gain a rich, in depth understanding of GP Practice Staff’s perceptions of the HOME BP programme.

Ethics approvals were obtained from NRES committee London-Fulham (13/LO/1502).

### HOME BP healthcare practitioner intervention

The HOME BP intervention for healthcare practitioners consists of a Prescriber’s guide and a Supporter’s guide. Table 1 provides an overview of the key tasks carried out by patients, Prescribers and Supporters within the HOME BP Intervention; the content of HOME BP is also reported in full elsewhere [8]. The Prescriber’s guide aims to enable GPs and nurse prescribers to efficiently prescribe medications for hypertension based on home blood pressure readings, with the aim of reducing clinical inertia.

**Table 1:** Events within HOME BP for Patients, Prescribers and Supporters

Event	Occurrence
Patients complete the first session of HOME BP Designed to raise patients’ motivation for making medication changes	At the beginning of HOME BP
Practitioners and patients meet for a baseline medication review Practitioners choose three medication changes which will be implemented later if the patients” blood pressure remains above target.	A week or two after the patient completes session 1
Patients complete the second session of HOME BP Which demonstrates how to monitor blood pressure at home.	After baseline review
Patients practice monitoring their blood pressure at home for a week and meet with their Supporter if help is required Supporters review patients’ practice blood pressure readings online and ask patients to do a further practice if they have experienced problems with this process. Patients can meet with their supporter if they have questions or concerns about home monitoring at this point.	After session 2
Patients monitor their blood pressure at home for 1 week every month If blood pressure remains above target for two consecutive months the patient requires a medication change and is informed by HOME BP.	After a week of practice monitoring has been completed. Then monthly for the remainder of the intervention.
Practitioners are alerted by email if patients require a medication change Practitioners create a prescription for the pre-agreed medication change, which patients can collect from reception or receive by post without requiring a consultation with the practitioner.	If a patients’ blood pressure remains raised for two consecutive months.
Supporters send patients a supportive email To encourage engagement with HOME BP and home monitoring or lifestyle changes.	8 weeks after the beginning of HOME BP, then every 4 weeks after this.
Patients are given access to online support with making lifestyle changes Patients can get help with changing their diet, increasing physical activity, reducing alcohol or losing weight. They are given access to this content 9 weeks after they begin HOME BP.	9 weeks after beginning HOME BP (to allow time for patients to get used to monitoring blood pressure)
Patients can meet with their Supporter To discuss the lifestyle changes that they might like to make. This appointment is optional.	10 weeks after beginning of HOME BP

The Prescriber’s guide aimed to overcome known barriers to prescribing medication for uncontrolled hypertension. We drew on Social Cognitive Theory, which views behaviour as determined by outcome expectancies, self-efficacy and the environment [11]. Known barriers to prescribing medication for uncontrolled hypertension can be mainly organised into unhelpful outcome expectancies (e.g. that prescribing might not be necessary or safe) and a lack of self-efficacy for prescribing in this context. HOME BP was therefore designed to foster more positive outcome expectations and build self-efficacy for prescribing. HOME BP also altered practitioners’ environments by creating automated emails which would alert practitioners to the need to prescribe if patients’ hypertension remained poorly controlled. Table 2 outlines known reasons for clinical inertia and an explanation of how HOME BP addresses these barriers to prescribing.

**Table 2:** How HOME BP addresses clinical inertia in prescribing hypertension medication

Reason for clinical inertia	HOME BP’s solution
Clinical inertia can occur because practitioners are not confident that the patient’s raised clinic reading is an accurate representation of their normal day-to-day blood pressure (e.g. could be white coat hypertension) [38] and so they expect that making a medication change might be unnecessary or unsafe.	Home BP overcomes this problem by enabling practitioners to base medication decisions on more reliable evidence – the mean of home blood pressure readings recorded every day for one week out of every month. If readings are above target for two consecutive months then this is strong evidence that a medication change is required.
Clinical inertia can also occur because practitioners are concerned that increasing medication may be disliked by patients and could negatively impact on the patient-practitioner relationship.	At the beginning of the HOME BP programme patients learn about the benefits of making medication changes if blood pressure is above target. After this patients meet with the practitioner to agree which medication changes would be most suitable if their blood pressure remains above target. The practitioner can therefore be assured that patients are in agreement with the practitioner’s decision to prescribe if they do need to implement a medication change.
Clinical inertia can also occur when practitioners are not sure which drugs to implement within a consultation with a patient whose blood pressure is raised [38].	Deciding medication changes in advance of their implementation gives practitioners more time to decide which medication changes might be most suitable and so may overcome this problem. Practitioners are shown brief modelled examples of a Prescriber choosing drugs for a patient in HOME BP, as well as guidance from NICE on choosing medications for hypertension [9].
A final important reason for clinical inertia appears to be clinicians not understanding treatment targets, or believing that the patient is ‘close enough’ when they are above target [5].	This is addressed in HOME BP by the programme emailing Prescribers to alert them when a patient’s blood pressure remains above target and medication change is required.

The second part of the HOME BP intervention for practitioners is the Supporter’s guide. This aims to enable nurses and healthcare assistants to provide two standardised support appointments to patients. The first is to help patients with any problems with monitoring their blood pressure at home. The second is to discuss potential lifestyle changes that patients might like to make to help control their hypertension. Supporters also send patients an email once a month to provide encouragement for home monitoring and lifestyle changes.

One problem faced by practitioners providing support for online interventions is that they often lack the knowledge or behavioural counselling skills to provide behaviour change support [12]. We designed the CARE approach (Congratulate, Ask, Reassure, Encourage) to be easy to deliver for practitioners to provide patient-centred care to support online interventions. In the CARE model, the intervention ingredients and behaviour change techniques are therefore delivered by the digital intervention (ensuring fidelity). The Supporter’s role in HOME BP is to provide the human support which can increase adherence to digital interventions [13, 14].

CARE was developed using an Evidence-, Theory- and Person-based approach [10, 15, 16], drawing on self-determination theory [17], evidence from the literature and our previous qualitative work with practitioners who have delivered similar approaches in our digital weight loss intervention [18]. The components of the CARE approach and their theoretical basis are outlined in Table 3.

**Table 3:** The CARE approach: Congratulate, Ask, Reassure, Encourage

Guidance given to Supporters about CARE	Theoretical Basis
Congratulate the patient on anything they did well. This can include taking part in the study, logging onto HOME BP, completing the first online session, monitoring their blood pressure at home or making healthy changes to their lifestyle. Example: “Well done for taking part in this study, I think it's great that you want to learn more about self-monitoring your blood pressure and have that extra control over your health".	Praise is focussed on the process of behaviour change (e.g. “well done for monitoring your blood pressure at home”, or “great job on sticking to your physical activity goal”), rather than the person as a whole (e.g. “you’re so good at cutting down on salt”). Process focussed praise can enhance autonomous motivation [39, 40], as well as feelings of competence and relatedness [40]. Praise is also informational (“That’s great that you’ve logged on and had a look at HOME BP”), rather than controlling (“Well done you’ve logged on to HOME BP, as you should”) which also supports autonomy [32, 39]. Participants who have not engaged with behaviour changes are not pressured, as minimising pressure supports autonomy [41].
Ask the patient how they are getting on, ask if they have any questions or concerns. If they have any concerns then you can ask them what solutions they would like to try - remember the aim is for people to become their own health trainer, not to rely on others. Example: “How have you been getting on with monitoring your blood pressure at home? How have you been finding it entering your readings on the HOME BP programme?"	Eliciting potential barriers and exploring possible solutions with patients can build more autonomous motivation [42]. This should also help patients to feel understood and cared for, which can enhance relatedness [42]. Emphasis is put on discussing the patient’s (rather than practitioner’s) ideas of possible solutions to challenges, to help build their feelings of competence and to help them to rely on themselves, rather than the practitioner, for solutions.
Reassure the patient about any concerns they have. Example: "It's really normal for your blood pressure readings to vary day to day, that's why monitoring your blood pressure regularly at home is so useful as it gives a much better indication of your average blood pressure than one reading in the Surgery".	Acknowledging the patient’s feelings can help support autonomy [40] Systematic review evidence indicates that cognitive reassurance (providing explanations and education) is associated with higher patient satisfaction, enablement and improved symptoms [43]. Reassurance is also associated with more patient centred care [44].
Encourage the patient to keep monitoring their blood pressure, entering their blood pressure readings into HOME BP, taking their medication and making any lifestyle changes that they discuss with you. Example: “It would be great if you can carry on monitoring your blood pressure when HOME BP sends you a reminder to do it. This will really help make sure we can find the right medication for you, and hopefully get your blood pressure to be better controlled".	Here practitioners provide non-controlling feedback, which can help support autonomy [40]. Practitioners provide a rationale for encouraging patients to continue with a behaviour change (e.g. how much it will help their health), as this can support autonomy [42] Encouragement is also associated with more patient centred care [44].

### Recruitment and procedure

Seven GP practices from the South of England took part, including practices from a mix of urban and rural settings. In total 55 Practice staff participated in the 7 focus groups, including General Practitioners (GPs; *n* = 16), practice nurses (*n* = 9), healthcare assistants (*n* = 6), reception staff (*n* = 17) and practice managers (*n* = 7). This allowed exploration of what the clinicians thought of our interventions as well as any wider issues with implementation of the intervention which might impact on, or be influenced by reception staff and practice managers. Each focus group consisted of staff at a single practice. Table 4 provides an overview of the staff involved in each focus group.

**Table 4:** Staff participating in focus groups

Focus group	Participating Practice Staff	
	Female	Male
1	1 GP, 2 nurses, 1 practice manager, 3 reception administrators	3 GPs
2	1 GP, 1 nurse, 1 practice manager, 1 reception administrator	3 GPs
3	1 GP, 1 nurse prescriber, 2 nurses, 2 HCAs, 1 practice manager, 3 reception administrators	
4	2 GPs, 1 nurse, 1 HCA, 1 practice manager, 5 reception administrators	
5	2 GPs, 1 nurse, 1 HCA, 3 reception administrators	1 practice manager
6	1 GPs, 1 HCA, 1 practice manager, 1 reception administrator	1 GP
7	1 nurse, 1 HCA, 1 practice manager,	1 GP, 1 reception administrator

Prior to the focus groups, staff were given access to HOME BP. Written consent was collected at the beginning of each focus group. Focus groups were carried out by KB (a health psychologist) and KM (a research assistant). The focus groups started off by exploring how each practice currently managed hypertension, followed by what practice staff thought of the idea of the HOME BP intervention supporting patients in monitoring their blood pressure at home and making lifestyle changes. Practice staff were asked what they thought of the Prescriber’s and Supporter’s guides, open-ended questions explored what they liked or disliked about these guides and how they would feel about implementing the procedures in their usual practice. The focus groups were audiotaped (median duration 45 min) and transcribed verbatim.

### Data analysis

An inductive thematic analysis [19] was conducted, to explore Practice staffs’ perceptions of HOME BP. Our Person-Based Approach involved paying particular attention to any barriers to behavioural changes (e.g. prescribing, providing support) or potential implementation of the HOME BP procedures. The data enabled modifications to be made to improve HOME BP to address concerns aiming to make the intervention more persuasive, acceptable and feasible to implement. This was an iterative process moving between data collection, analysis, modifications to the intervention and then further data collection. Occasionally we waited to collect feedback in multiple focus groups before making a change to the intervention, in other cases it was clear to see after a single focus group that a change was needed. Sometimes we found it useful to discuss a possible modification to the intervention with practitioners in the focus groups before implementing it in HOME BP, to help assess whether it might be acceptable. Saturation was deemed achieved as staff did not raise important new concerns or challenges to the acceptability or feasibility of the intervention in later focus groups.

The analysis was initially carried out by KB, an experienced qualitative researcher. First the researcher listened to, read and re-read the focus group transcripts. All data relating to the research question (‘how do practice staff perceive the HOME BP intervention?’) was coded. Whilst this meant that the vast majority of the data was coded, data which was not relevant to the research question (e.g. discussion of study procedures such as participant information sheets or speculation about study uptake) was not coded. A coding manual was created based on initial coding. Constant comparison was used and the coding manual was continually refined and updated to ensure that codes were used consistently and accurately reflected the data [20]. Codes which identified similar aspects of the data were clustered together into themes. An audit trail and memos were maintained throughout the analysis. The final codes, themes and modifications to HOME BP were agreed between KB, KM and LY. Deviant cases, which diverged from the dominant trends, were identified to help consider the limits of the analysis and to ensure that no data were overlooked.

## Results

Three themes were identified: ‘Managing blood pressure at home’, ‘Agreeing medication changes in advance’ and ‘Supporting patients with HOME BP’. These are discussed in detail below and an overview of the themes and codes is provided in table 5. Practitioners’ feedback was also used to make modifications to the HOME BP intervention to improve its acceptability and feasibility to implement in practice; these changes are described below and an overview is provided in Table 6.

**Table 5:** Themes and Codes identified within analysis

Theme	Codes
Managing blood pressure at home	Home monitoring an empowering process
	Home monitoring overcomes the problem of clinical inertia
	Home monitoring could save (or cost) time
	Patients may get obsessed with monitoring their blood pressure
	Home monitoring might be anxiety provoking
	Usual practice for managing high blood pressure
	Currently no system for recording home readings in practice
	Are home readings accurate?
	Home monitoring overcomes problems of white coat hypertension
	Useful that home readings are emailed to practitioners
	A system for responding to emails from HOME BP
Agreeing medication changes in advance	Understanding medication changes in advance may be empowering for patients
	Concerns about choosing medication changes in advance
	Potential solutions to problems with choosing medication changes in advance
	Does the baseline medication review need to be longer to allow explanation of medication changes?
Supporting patients using HOME BP	Useful that HOME BP provides support with behaviour change
	Supporters role with behaviour change viewed as important
	Supporter’s guide accessible
	Practitioners value building patient autonomy (avoiding dependence on practitioners)
	Perceptions of non-directive support using CARE
	Perceptions of congratulating patients using CARE
	Perceptions of reassuring patients using CARE
	Desire to see the patient intervention
	Lack of time to provide support

**Table 6:** Modifications made to HOME BP based on focus group feedback

	Focus group feedback	Changes made to HOME BP
1	Concerns that patients monitoring their blood pressure at home might contact the practice more, because of concern about their readings.	TASMINH2 [6] did not find that patients monitoring their blood pressure at home consulted more frequently than those in usual care. This information was added to the Prescriber’s and Supporter’s guides to reassure practitioners that this is unlikely to be the case.
2	Concerns about the accuracy of home blood pressure readings, particularly very high readings.	An explanation was added which described the procedures employed to ensure that patients’ readings would be accurate. This includes patients completing a week of practicing monitoring their blood pressure before beginning to monitor it for real. Patients can email their practice readings to their Supporter for feedback. They can also meet with their Supporter if they experience problems with home monitoring, or have concerns about their readings. It was also explained that few patients in the TASMINH2 study got very high readings [6], indicating that this is unlikely to be a regular occurrence.
3	Concerns about choosing 3 drugs in advance. This concern was based on: 1- Not knowing which drugs to pick and a concern that there might not be enough drugs to choose from. 2- Concerns about interactions between drugs in combined medication regimes.	1- To address the first concern we added explanation that medication changes could include increases in drug doses, not just adding further drugs. We also included a scenario of a complex patient taking 3 drugs, showing 3 possible medication changes which could be suggested for the patient in the first instance and a further 3 which could be used if the first 3 were unsuitable. 2- To address the second concern we showed Prescribers evidence of the safety and efficacy of this approach. We presented the findings of the TASMIN-SR study [7], which found that patients with co-morbidities who were already taking multiple drugs did not have more side effects (but did significantly reduce their blood pressure) compared to those receiving usual care when they monitored their blood pressure at home and implemented pre-agreed medication changes when blood pressure remained raised. The Prescriber’s guide also reminded prescribers to check the patients’ notes to ensure a pre-agreed medication change was still appropriate.
5	Two GPs wanted the baseline medication review to be longer, others disagreed.	The information was updated to suggest that some practitioners might find it helpful to use a double appointment for medication reviews for their first patient in the intervention group, to allow time to get used to the study procedures, but that after this a single appointment should suffice.
6	Nurses at the first focus group were concerned that they need to give patients advice, as patients would expect this.	Information was added to reassure Supporters that the CARE approach (without giving advice) has been used successfully in previous studies. Quotes from patients and practitioners were shown, which demonstrated the acceptability of this approach.
7	Nurses at the first focus group were also concerned that they wouldn’t know how to congratulate patients who demonstrated a lack of adherence, or reassure patients about their concerns.	Detailed examples of how to congratulate and reassure patients were added to model this approach.
8	Most Supporters wanted to be able to view the patient website	This was made available to Supporters, with an explanation that it was not necessary to memorise this information, since their role would be to provide support using the CARE model, not specific advice.
9	A few nurses noted that a lack of time might be a barrier to providing support.	Patients are offered two, optional, ten minute appointments during the 12 month study. It is likely that not all patients will choose to attend these appointments (this has been the case in our other web-based interventions, e.g.[14]). Nevertheless, some practices may find this time commitment too great. However, we decided to keep these support appointments as similar interventions have larger effects if human support is provided. The majority of support for patients is provided by email, with emails that are pre-written and only need to be tailored briefly to the patient, meaning this support should be very quick and easy to deliver.

### Managing blood pressure at home

Many practice staff viewed HOME BP as having the potential to empower patients to self-manage their own hypertension. Some practitioners believed this could lead to better patient adherence.

*“A great idea and I think that anything that can get the patients looking after their conditions themselves is to be encouraged because if they manage it themselves they’re more involved, they see it more as a, you know, important and hopefully we’ll get better controlled blood pressures”. Nurse prescriber, FG3*

Deviant case analysis highlighted that one GP acknowledged that enabling patients to monitor their blood pressure at home might overcome clinical inertia, particularly in situations where the clinician had failed to prescribe because of time constraints within consultations. It was perhaps unsurprising that most GPs did not talk about this, since admitting to clinical inertia might be considered sub-optimal practice.

*“(Blood pressure) can be tacked on at the end of a very long consultation…that’s one of the reasons why if it’s a few pips above normal we might not be as pro-active as we could be. And then before you know it it’s another three months for another check and then we’ll see it again. Whereas I like the idea this is once a month you know and (the patient is) taking charge of it”. GP FG5*

Some GPs and nurses believed that the home monitoring procedures would save them time as medication changes would be dealt with remotely, rather than within a consultation. However, others felt that increased home monitoring could mean that patients “*may get in touch with us more frequently which means taking up more of our time”. (GP, FG6)*. These practitioners were often concerned that patients might get obsessed with monitoring their blood pressure, or become anxious when they get high readings, which could be a disempowering process for the patient and time consuming for the practice.

*“Some (patients) get obsessed with monitoring, if it’s high* then they do it again…*I saw one patient took it twenty-eight times in a day… So I don’t think obviously it’s ideal for everybody but you’ve got to highlight, find those patients who do get extremely anxious if it’s slightly high and who will lie in bed all day”. Nurse, FG3,*

Most practices were already working with patients who sometimes monitored their blood pressure at home, but lacked a robust system for responding to home readings.

*“Some of our patients are doing it essentially anyway but there’s no avenue to feed back so they’re doing the recordings and then they’re just looking and then panicking or not panicking or doing nothing so (HOME BP) will be quite good”. GP, FG5*

A few GPs, nurses and HCAs had concerns about the accuracy of home blood pressure readings, particularly very high readings and whether they could be trusted. However, other practitioners were used to patients monitoring at home and then prescribing based on these readings. Others felt that home readings would probably be more accurate, particularly in cases of white coat hypertension.

We employed several strategies to ensure that patients’ readings would be accurate and in response to practitioners’ concerns about this we added an explanation of these strategies to the Prescriber’s guide (see Table 6, point 2 for details). Practitioners in later focus groups noted that these strategies would likely ensure the accuracy of home readings.

All practice staff discussed the email alerts that would be sent from HOME BP to alert practitioners when home readings indicated a medication change was necessary. Some clinicians were concerned that their practice might not have a system to deal with emails, but receptionists and practice managers at all practices reassured practitioners that generic email addresses already existed and were regularly checked, which would enable easy implementation of HOME BP email prompts. Many practitioners saw emails about patients who were self-monitoring as a useful tool for managing patients, which would save both the practitioner and patient time.

*“It’s an organisational shift that we’re going to need to make at some stage soon anyway. I’ve been doing it for a number of years but we haven’t had a proper system set up…It’s undoubtedly much more time efficient”. GP, FG2*

Deviant case analysis showed that one practice was concerned about the quantity of emails that they might receive, anticipating that their GPs would receive numerous emails from patients, which based on our previous web based studies and our initial pilot work with HOME BP we think is very unlikely to be the case.

### Agreeing medication changes in advance

Some practitioners were very positive about the prospect of choosing medications to be implemented later, if blood pressure remained uncontrolled. They felt that agreeing these medications with the patient at a baseline appointment would help empower the patient to self-manage their hypertension.

*“It’s a brilliant idea… People are going to be aware of, actually, why am I doing this? What’s the importance? What are the steps? What happens next? That’s all upfront. A lot of the time people feel disempowered. You come to the GP, you have your blood pressure taken. If it’s raised they scratch their head and put you on some medication, tell you to come back…Whereas this is right at the opposite end of the spectrum”. GP, FG2*

In contrast, some GPs were concerned about picking 3 medication changes in advance. A few felt that they would be unsure of which drugs to pick. Others were concerned that in the time between agreeing medication changes and these being implemented the patient might receive a drug for another health condition which could interact with the previously agreed medication changes, raising the potential for harm from interaction effects.

*“I’d find that quite difficult because it’s quite a lot to think about if you’ve already got somebody who’s got pre-existing hypertension on a few agents to then think about three (medication changes) – I mean I’d probably struggle to think of two to be honest”. GP, FG6.*

*“Pre-empting what medicines to use is potentially fraught with danger because they may have been seen for something else and in the meantime been given another drug which interacts with what you originally said”. GP, FG1*

GPs suggested that a potential solution might be to prompt them to check the patient’s medication list when implementing a pre-planned medication change, to check for possible interactions, which was subsequently incorporated into HOME BP. The other ways in which we addressed practitioners’ concerns about choosing three drugs in advance are outlined in point 3 of Table 6. These changes appeared successful, since practitioners in later focus groups who saw our updated Prescriber’s guide did not mention these concerns.

A few GPs believed that patients with uncontrolled hypertension would be hard to choose three drugs for, as they would likely have tried drugs before and experienced side effects, meaning the pool of drugs to pick from would be smaller.

Deviant case analysis highlighted that two GPs were keen for the initial medication review to be longer, to explain the three planned medication changes to patients. Other clinicians felt that they would only need 10 min. Based on this deviant cases analysis we re-designed our initial medication review procedures to ensure acceptability for all prescribers (see table 6, point 5).

### Supporting patients using HOME BP

Practitioners were pleased that patients would get support with lifestyle changes through HOME BP, as they often had limited time to give advice about this. GPs liked the fact that nurses or HCAs would provide care at critical time points to support behavioural changes.

Nurses and HCAs generally responded positively to the Supporter’s guide, noting that it was *“easy to understand”.* (FG7, HCA), and valued its approach of building patient autonomy, rather than reliance on the nurse.

“I like the word ‘Supporter’ actually, not, you know, not nurse or you know because (patients) do expect you to come up with a magic wand at times”. Nurse, FG5

The Supporter’s role in HOME BP was aimed at providing non-directive care to facilitate the building of autonomous motivation for behavioural changes. Supporters were asked not to give advice, but to follow the CARE approach, discussing the patient’s own ideas for solutions to problems. The first focus group elicited negative perceptions of CARE as nurses were concerned that it would be difficult not to give advice, as they were used to doing this in their daily roles, and felt patients would expect this.

*Nurse 1: “I suppose it’s our role, you know, within a consultation is to give advice and talk to people about what they’re doing and how they can change what they’re doing. And if you’re just sitting there saying, “Well you’ll do this, oh I think you better go back to the website and have a look and see what it tells you to do”.*

*Nurse 2: “Could make them feel unsupported”. FG1*

We realised at this point that telling the nurses to provide CARE, without providing a clear rationale for why this was a useful approach was probably unpersuasive and perhaps a challenge to nurse’s autonomy. We amended the Supporter’s guide to include information to reassure Supporter’s that CARE (without giving advice) has been used successfully in previous studies. We also added quotes from patients and practitioners, which demonstrated the acceptability of this approach. We hoped that this evidence would help practitioners to buy into CARE, rather than feeling they had been told to do it and that this would help persuade practitioners to try it out. After making these changes, practitioners in later focus groups did not raise concerns about CARE or not being able to give advice. Instead they were very positive about the approach, indicating that our changes had been successful. Some practitioners even felt that CARE could reduce consultation times.

*“I like the idea that we’re not supposed to give them all the answers, so that they’re meant, we just redirect them and support them actually, I think that’s really, really positive from our point of view … it’ll reduce consultation times, absolutely in the long run”. Nurse FG5*

Some nurses and HCAs also viewed CARE as very similar to their normal approach.

*“I think it’s probably work that everybody sat round the table is doing already”. Nurse, FG2*

Another concern about CARE raised by the nurses in focus group 1 was that they could not easily congratulate patients who had not made good progress. They also felt they would struggle to reassure patients without having more information about how to do this.

*“When people come in and you’re saying, “Oh well done,” I just, I don’t feel terribly comfortable doing it. It’s just, it’s sort of too, for me, it’s just too, “Oh right, now I’ve got to congratulate you and I’ve got to do this…” I just, I prefer just to be easy to say what I want to say…It’s assuming there is something positive”. FG1*

Detailed examples of how to congratulate and reassure patients were therefore added to the Supporter’s guide to model this approach. Supporters in later focus groups did not appear concerned about congratulating or reassuring patients, indicating that this change was likely to have been successful.

Some nurses wanted to view the pages that the patient saw in order to provide adequate reassurance to patients. These pages were therefore made available to Supporters, with an explanation that it was not necessary to provide this information, since their role would be to provide support using CARE, not specific advice.

“*Can we have a look at (the patient) site? Because only if people are coming in saying “Oh, well I’ve read this” And phew, I*’*ve never seen it so I don*’*t know”. HCA FG7*

A few nurses and HCAs discussed that lack of time was a barrier to providing face-to-face or telephone support. A description of how we responded to this concern is outlined in point 9 of Table 6.

## Discussion

Overall, primary care staff indicated that the HOME BP intervention appeared acceptable, engaging and persuasive. However, staff also had some concerns. Our iterative, Person-Based Approach [10] meant we were able to address these concerns with modifications to HOME BP. Below our findings are discussed in relation to the wider literature.

### Relating findings to the wider literature

#### Managing blood pressure at HOME

Practitioners noted that HOME BP would empower patients to play a more active role in self-managing their blood pressure. However, a perceived problem was that patients might become obsessed with monitoring their blood pressure, or overly anxious about their readings. Practitioners have expressed these concerns elsewhere [21, 22], yet evidence suggests that patients do not become preoccupied with monitoring their blood pressure [23], or exhibit increases in anxiety when monitoring their blood pressure at home [6]. In the context of over-stretched primary care services practitioners in the current study were also concerned that high readings might lead to additional consultations, but evidence from TASMINH2 indicates that few patients have very high readings and that the study procedures do not lead to more consultations than usual care [6]. This information was added to the Prescriber’s guide to provide reassurance.

#### Agreeing medication changes in advance

Some practitioners viewed agreeing medication changes in advance as empowering the patient by informing them of how their future blood pressure treatment would work. However, others had concerns about this approach, including a lack confidence in choosing multiple medications and concerns that pre-agreed medication changes might be dangerous. Implementation theory, such as Normalisation Process Theory (NPT), would suggest these perceptions could be a barrier to enacting the procedure for intensification of treatment (i.e. a challenge to collective action) if left unaddressed [24, 25]. Previous studies have also found that GPs may sometimes lack the skills needed to combine multiple medications and that they have concerns that combining medications may be dangerous [26], particularly in patients with co-morbidities [27, 28]. However, appropriate combination therapy (combining multiple medications) is recommended in national guidelines for the management of hypertension [29] and controlling hypertension is often particularly important in patients with co-morbidities (such as diabetes and Chronic Kidney Disease) [29]. Modifications to the Prescriber’s guide, including reassurance about the efficacy and safety of these procedures (as proven in the TASMINH studies) [6] and examples modelling how to choose medications for complex patients, should provide reassurance to practitioners that appropriate combination therapy can be achievable and safe. Process interviews with practitioners who chose medications in advance in the TASMINH2 study did not highlight a lack of confidence in choosing multiple drugs, or perceptions that combination therapy might be dangerous [30], so it may be that when practitioners implement the procedures in practice they overcome their initial concerns. It will be important to explore these issues further in process interviews when practitioners have had time to try out the procedures in the HOME BP trial.

#### Supporting patients with HOME BP

Nurses in focus group 1 felt that Supporters should provide advice during HOME BP support sessions, as patients would expect this. These nurses appeared to see it as *their role* to provide advice, rather than non-directive support. This was an important potential barrier to implementation since both NPT [24, 25] and evidence from a review of reviews [31] indicates that in order for healthcare professionals to buy into a new intervention they need to view their role in the intervention as congruent with their perceived role in their wider work. We realised that nurses might be more willing to try out a new practice if they felt that they were choosing to do so, rather than just being told to. Since providing a rationale for behaviour change can support autonomy [32] we added evidence of the acceptability and effectiveness of CARE to HOME BP, including quotes from nurses and patients who had used or received an earlier version of this approach in our previous studies [13, 18, 33]. Supporting nurses’ autonomy in this way seemed to work well, as in later focus groups no nurses raised concerns about not being able to give advice and many felt that the approach was either close to how they already worked, or a valuable way of empowering patients to get more involved in their own healthcare.

Most practice staff viewed the procedures involved in the CARE approach as potentially easy to put into action. However, there were also some challenges, for instance, nurses at the first focus group were not confident in how to reassure or congratulate patients using CARE. It seemed likely that the addition of further examples of how to enact the CARE approach, which were added to the Supporter’s Guide, overcame this barrier to implementation, as no practitioners in later focus groups mentioned similar concerns and all viewed CARE as easy to implement. Human support is often viewed as important within digital interventions, as it can boost motivation and engagement [14]. The CARE approach may be useful in other interventions which provide human support alongside a digital tool, as CARE enables human support to be provided without compromising the fidelity of advice or behaviour change techniques which are delivered solely through the digital tool. CARE also means that healthcare practitioners don’t need to be skilled in behavioural counselling techniques, which enables a broader range of staff to deliver human support. Our initial work with patients indicates that the CARE approach is liked by patients [33], however, further research confirming the acceptability and effectiveness of the CARE approach would be useful.

A few nurses reported that they might not get time to offer HOME BP patients two (optional) support appointments per year. In terms of the actual impact on staff time, even if all patients attend these two optional appointments, plus their baseline medication review appointment, they would still only receive three hypertension appointments per year. This is comparable with the current average seen in usual care [6], and if the procedures lead to the same improvements in blood pressure as in the TASMINH2 study then the intervention would very likely be cost-effective. Still, the current financial pressures on primary care may mean that some practices may feel unable to provide this small amount of support to patients.

#### Implications for intervention development

Adopting a Person-Based Approach to intervention development in this study helped identify and seemingly overcome potential barriers to future implementation of the HOME BP intervention, increasing the acceptability and persuasiveness of this intervention Identifying the key context-specific barriers to implementation is important given the wider literature, which indicates great variation in the effectiveness of interventions designed to alter primary care practitioners’ behaviour (and subsequent patient health outcomes) [34]. Many interventions have only modest effects and some no effect at all [35, 36]. Evidence indicates that such poor effects are often due to a number of problems found in the primary care setting; barriers to implementation include professional (e.g. narrow definition of role as only biomedical), organisational (e.g. limited resources) and contextual (e.g. financial incentivisation of some behaviours and not others) [31, 37]. While theory and previous research helped us to anticipate many factors that might influence implementation of our intervention, the inductive approach used in this study highlighted those issues of particular importance to our target users, which we were unable to predict from our prior behavioural analyses. It is for this reason that the Person-Based Approach uses inductive qualitative research iteratively, at the development as well as planning stage, allowing modifications to be made to optimise a prototype intervention before it is implemented.

#### Limitations

This study was able to explore practitioners’ perceptions of the HOME BP intervention, but not their experiences of enacting the HOME BP procedures in practice. Exploring these experiences in qualitative process studies embedded within the pilot trial of HOME BP will provide a vital next step in ensuring the successful implementation of this intervention. Another limitation of this study was that interviews were conducted by researchers who were involved in the development of HOME BP, which might have led some staff to provide socially desirable answers. However, this seemed less likely, since staff were willing to express negative views. We were able to sample an even balance of male and female GPs, but the majority of reception staff and practice managers and all of the nursing and healthcare assistants were women. Whilst this reflects the reality of normal practice for most of these staff groups, it is possible that men’s views might differ in some way, although it is useful to note that views did not vary based on gender within the GPs sampled.

## Conclusion

This paper provides a demonstration of using the Person-Based Approach to develop a digital intervention, which may be of use to others developing interventions. The study provides detailed insights into practitioners’ perceptions of the HOME BP intervention and how such an intervention can be modified to address concerns. Some potential challenges to implementation were identified, including concerns about the accuracy of patients’ home blood pressure readings, a lack of confidence around combining medications and perceptions that the CARE approach was not congruent with practitioners’ perceived roles. Modifications to the intervention appeared to have helped overcome these concerns and improved the acceptability and feasibility of implementing HOME BP in practice. Research is now needed to explore the effectiveness of HOME BP, as well as to explore practitioners’ experience of implementing the HOME BP procedures in process analyses, which will provide further insight into whether the HOME BP intervention will be easy to implement in practice.

## Abbreviations

NPT: Normalisation process theory
